# Epithelial-myoepithelial carcinoma in the ventral surface of the tongue

**DOI:** 10.1590/S1808-86942010000400023

**Published:** 2015-10-19

**Authors:** Felipe Rodrigues de Matos, João Luiz de Miranda, Ana Teresinha Marques Mesquita, Cássio Roberto Rocha Santos, Roseana de Almeida Freitas

**Affiliations:** a DDS. MSc. Student in Oral Pathology; b PhD in Oral Pathology – Professor at the Federal University of the Jequitnhonha and Mucuri river valeys; c MSc in Stomatology, Professor at the Federal University of the Jequitnhonha and Mucuri river valeys; d PhD in Maxillo-Facial Surgery, Professor at the Federal University of the Jequitnhonha and Mucuri river valeys; e PhD in Oral Pathology. Professor of the Graduate Program in Oral Pathology – Federal University of Rio Grande do Norte

**Keywords:** salivary glands, tongue, tongue neoplasms

## INTRODUCTION

Myoepithelial-epithelial carcinoma (MEC) is a low malignancy grade neoplasia which has a prevalence of 1% to 2% among all primary tumors of the salivary gland^1^. It has a higher incidence at 60 years of age. About 60% of the patients are females. The parotid gland is the most often reported anatomical site; nonetheless, other sites have been reported, such as the submandibular gland, minor salivary glands, paranasal sinus, trachea, lacrymal gland and nasal cavity^1^^,^^2^^,^^3^. Rare are the instances in which this tumor involves the tongue1. Clinically speaking, the well outlined swelling is the only signal, associated or not with pain^1^.

Microscopic findings reveal a solid tubular growth which is usually made up of a group of internal epithelial cells with eosinophylic cytoplasm and another group of external myoepithelial cells (MC) with clear cytoplasm^1^^,^^4^^,^^5^.

## CASE REPORT

**Figure 1:**
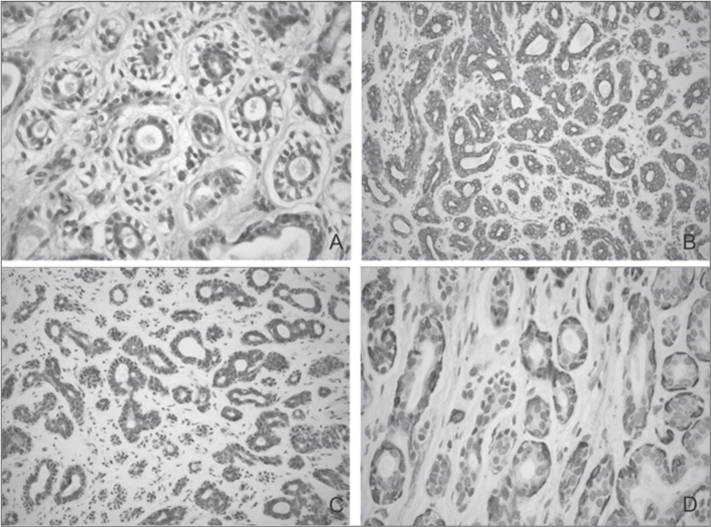
Histopathology findings - (A) Parenchyma with ductiform structures, made up of internal cells with eosinophylic and cubic cytoplasm and the external layer made up of clear cells - HE; (B) Immunopositiveness for CK 7 - SABC; (C) Immunopositiveness for CK 8 - SABC; (D) Imunnopositiveness for SMA - SABC.

A 48 female, Caucasian patient came to our stomatology clinic on September of 2002, complaining of a growth on her tongue. During the interview she reported she had been feling a discomfort in the region for a month. During intraoral examination we noticed a normal-looking mucosa, with normal color, without any visible change. However, during palpation we felt an endophytic nodular, soft lesion, measuring approximately 1cm. The diagnostic hypothesis was lypoma. We did an excisional biopsy. During microscopy with HE dye, we noticed that the specimen had ductiform structures in its parenchima, made up of internal cells with eosinophylic and cubic cytoplasm and na external layer made up of clear cells (Fig. 1A). In the immunohistochemistry, the internal cells of th ductiform structures were positive for cytokeratin (CK) 7 (Fig. 1B) and 8 (Fig. 2C), thus being epithelial cells. The external cells were reactive to the smooth muscle actin (SMA) antibody (Fig. 2D), showing the phenotype of myoepithelial cells. The histopathological diangosis was of MEC. The patient was then referred to the Oncology Clinic, and the physician decided for not doing another surgery, but rather to observe her. Nonetheless, the patient had a recurrence on August of 2006, four years after the first surgery and she was submitted only to a new surgery with safety margins. In three years of follow up there was no recurrence noticed.

## DISCUSSION

MEC is a rare malignant tumor of the salivary gland^1^^,^^2^^,^^3^. The patient is 48 years old, which is below the mean age of incidence^3^. Clinically, it can happen both in the minor as well as in the greater salivary glands^2^^,^^3^. The case here deals on a tongue belly MEC, which are rare in this site^1^. Localized swelling is the only clinical sign, which can lead the physician towards a diagnosis of a benign^3^ or malignant1 lesion. So far, there are no reports on the association of risk factors such smoking, alcohol and radiations in the development of MEC^1^^,^^3^.

The MEC diagnosis is based on the light microscopy and was confirmed by immunohistochemistry^1^^,^^4^^,^^5^. In this cases, the microscopic findings were important as per described in the literature.

As far as treatment goes, most of authors agree on surgical excision with proper safety margins, probservation and, in some cases, adjuvant radiotherapy when there is difficulty in outlining the surgical margins ^1^^,^^3^^,^^6^. Because of the very rarity of the lesion, we do not have a clear cut protocol for recurrencies, and the only thing that is done is a new surgical intervention with patient close follow up^1^^,^^3^. In the case hereby presented, we did an excisional biopsy and four years after the first procedure the patient developed a recurrence, and the second procedure was done with proper safety margins.

## FINAL REMARKS

It is mandatory to be aware of cases, such as the one presented here, so as not to underestimate tongue lesions and include neoplasia among differential diagnosis so that the surgical approach can be as accurate as possible. In cases of recurrence, te patient must be educated on the fact that it is a rare lesion which needs long follow up.
